# Motor Imagery Multi-Tasks Classification for BCIs Using the NVIDIA Jetson TX2 Board and the EEGNet Network

**DOI:** 10.3390/s23084164

**Published:** 2023-04-21

**Authors:** Tat’y Mwata-Velu, Edson Niyonsaba-Sebigunda, Juan Gabriel Avina-Cervantes, Jose Ruiz-Pinales, Narcisse Velu-A-Gulenga, Adán Antonio Alonso-Ramírez

**Affiliations:** 1 Centro de Investigación en Computación, Instituto Politécnico Nacional (CIC-IPN), Avenida Juan de Dios Bátiz Esquina Miguel Othón de Mendizábal Colonia Nueva Industrial Vallejo, Alcaldía Gustavo A. Madero, Ciudad de Mexico C.P. 07738, Mexico; 2 Institut Supérieur Pédagogique Technique de Kinshasa (I.S.P.T.-KIN), Av. de la Science 5, Gombe, Kinshasa 3287, Democratic Republic of the Congo; eniyonsaba@isptkin.ac.cd (E.N.-S.); pinales@ugto.mx (J.R.-P.); 3 Telematics and Digital Signal Processing Research Groups (CAs), Department of Electronics Engineering, Universidad de Guanajuato, Salamanca 36885, Mexico; avina@ugto.mx; 4 Institut Supérieur Pédagogique de Kikwit (I.S.P. KIKWIT), Av Nzundu 2, Com. Lukolela, Kikwit 8211, Democratic Republic of the Congo; 5 Instituto Tecnológico Nacional de México en Celaya (TecNM-Celaya), Av. Antonio García Cubas Pte 600, Celaya C.P. 38010, Guanajuato, Mexico; d2203002@itcelaya.edu.mx

**Keywords:** electroencephalogram, motor imagery, EEGNet, NVIDIA Jetson TX2, brain–computer interface, HaLT dataset

## Abstract

Nowadays, Brain–Computer Interfaces (BCIs) still captivate large interest because of multiple advantages offered in numerous domains, explicitly assisting people with motor disabilities in communicating with the surrounding environment. However, challenges of portability, instantaneous processing time, and accurate data processing remain for numerous BCI system setups. This work implements an embedded multi-tasks classifier based on motor imagery using the EEGNet network integrated into the NVIDIA Jetson TX2 card. Therefore, two strategies are developed to select the most discriminant channels. The former uses the accuracy based-classifier criterion, while the latter evaluates electrode mutual information to form discriminant channel subsets. Next, the EEGNet network is implemented to classify discriminant channel signals. Additionally, a cyclic learning algorithm is implemented at the software level to accelerate the model learning convergence and fully profit from the NJT2 hardware resources. Finally, motor imagery Electroencephalogram (EEG) signals provided by HaLT’s public benchmark were used, in addition to the k-fold cross-validation method. Average accuracies of 83.7% and 81.3% were achieved by classifying EEG signals per subject and motor imagery task, respectively. Each task was processed with an average latency of 48.7 ms. This framework offers an alternative for online EEG-BCI systems’ requirements, dealing with short processing times and reliable classification accuracy.

## 1. Introduction

Applications based on Brain–Computer Interfaces (BCI) are numerous in the recent literature due to their benefits in various domains [1]. Typically, BCI systems use brain signals to allow effective communication between a given user and local surroundings. BCI-based electroencephalographic signals (EEG) are the most implemented because of recent advances in brain electrical functioning studies and reliable technologies [2,3]. Such EEG signals were used by Fraiwan et al. [4] to evaluate the subjects’ enjoyment and visual interest in experiencing museum expositions. For instance, BCI-based EEG signals are used in biomedical applications for mental and cognitive disease diagnoses and rehabilitation [5,6]. Lastly, Hekmatmanesh et al. [7] proposed a systematic review of terrestrial and aerial Brain–Controlled Vehicles (BCVs) based on EEG, Electrooculographic (EOG), and Electromyographic (EMG) signals. Commonly, for BCI-based control systems, EEG signal patterns such as Steady-State Evoked Potentials (SSVEP) [8] and their variants are converted into commands to control wheelchairs, drones, prostheses, and arm robots, to cite a few.

Motor Imagery EEG signals (MI–EEG) are more interesting for EEG–BCI systems because the subject under test voluntarily generates such signals; thus, it can be used to control external applications [9,10] or for medical research [11,12]. Recent advances in developing BCIs based on MI signals focus on improving classification accuracy while reducing processing time and processing-unit computational resources [13,14]. This prevailing tendency is mainly motivated by online application requirements in robotics and specialized medicine to complete accurate and brief tasks satisfactorily [15]. In this sense, Huang et al. [16] controlled an integrated wheelchair robotic arm implementing a hybrid BCI based on EEG and EOG signals. Therefore, the robotic arm and wheelchair applications needed both high-accuracy classifications of left- and right-hand MI tasks and system portability to complete reliable actions. In another study, Al-Nuaimi et al. [17] implemented a controlled drone-based P300 BCI for military use, dealing with high accuracy, brief processing time, and BCI portability.

Concretely, numerous methods are used in the literature to conjointly address short processing time, reliable accuracy, and portability challenges. In this sense, channel selection -based strategies aim to process signals from a few discriminant electrodes instead of using all electrodes, reducing data size and processing algorithms’ complexity and, consequently, the processing time. For example, Moctezuma et al. [18] proposed the Non-dominated Sorting Genetic Algorithm II in the emotion recognition BCI with wearable EEG systems, selecting a set of 8–10 EEG channels instead of the 32 available. In parallel, various techniques based on deep learning offer satisfactory results without EEG signal preprocessing or implementing complex learning acceleration algorithms. Such an approach reduces the processing time significantly depending on the implemented neural architectures [19,20]. Other strategies typically work on hardware-based levels using traditionally powerful processing units despite the robustness of reliable data and algorithms [21,22].

Fortunately, advances in hardware re-configurable design technology have enabled the development of embedded electronic boards with powerful computing resources [23]. Those embedded boards are microcomputers generally supporting complex data processing, ensuring portability and reduced signal processing time because of dedicated core resources. Meanwhile, Majoros and Oniga [24] implemented a MI–EEG classifier based on a deep learning architecture for BCI applications on a Field-Programmable Gate Arrays (FPGA) card. Their work achieved an accuracy of 97.7%, classifying imagined tasks of opening and closing fists or feet into three classes; the neutral task was included. Further, Dabas et al. [25] used the Arduino Uno board to classify hand-gripping MI trials from channels C3 and C4 using the Support Vector Machines (SVM) classifier.

On the other hand, deep learning architectures have proven to have high performance as EEG signal classifiers in recent works, especially the compact convolutional neural network for EEG-based BCI (EEGNet) and its variants proposed by Lawhern et al. [26]. In this sense, Zhu et al. [27] developed an ensemble learning coupled to the EEGNet network to improve the ear-EEG signals’ classification for SSVEP-based BCI, achieving an accuracy of 81.74%. Lastly, Feng et al. [28] implemented a real-time EEGNet classifier on an FPGA board, using only 2.54% of the board’s resources and consuming 3.66% of the maximum power available. Similarly, Tsukahara et al. [29] achieved an accuracy of 88.75%, implementing the EEGNet architecture on a Virtex-7 FPGA platform to classify EEG data from the MNE dataset.

This work develops an embedded MI tasks classifier for BCI systems based on the EEGNet network by using the NJT2 board. The framework develops a subject-dependent classification approach, where data from each subject are processed separately. Therefore, the MI movements to be classified are the tongue, passive, left and right hands, and left and right legs. In the first step, the Accuracy Rating-based Classifier method (ARbC) and Channels Mutual Information-based Approach (CMIbA) are developed to make up discriminant channel subsets. Next, MI signals from discriminant channels are processed to be classified into the six aforementioned classes using the EEGNet network.

The main contributions of this paper are summarized as follows,

1. Results comparison of channel selection between the ARbC method and CMIbA.
2. Reliable accuracy results of the tongue, passive, left and right hands, and left and right legs MI tasks classification.
3. Processing time reduction using the NJT2 platform resources.
4. Convergence acceleration of the learning process implementing the Cyclic Learning Rate (CLR) algorithm.

In sum, this work deals with processing time reduction and reliable classification accuracy for embedded EEG BCI-based applications.

## 2. Related Works

In the recent literature on embedded BCIs (EBCI) based on MI–EEG signals, numerous works dealing with brief processing time and high classification accuracy have been proposed [30,31]. Embedded platform-based BCI designs aim to build low-cost and low-power consumption systems, meeting user adaptability and dedicating available resources to application-specific functions. Belwafi et al. [23] proposed a review of EBCI systems focusing on pathological disorders, functional substitution, and most implemented architectures. Despite recent advances in embedding computational architectures design, they reported a few of the EBCI systems presented in the related literature.

Generally, the central processing unit of the EBCI is ported by a microprocessor or microcontroller integrated into FPGA cards, Arduino boards, Nvidia’s developer cards, or specifically dedicated platforms. In this sense, Ma et al. [32] implemented a classifier-based convolutional neural network into a Xilinx FPGA platform to classify MI–EEG signals. Comparatively, implementing the same model on a portable computer equipped with the NVIDIA GeForce GTX1070 i7-7700 resources, the configured FPGA was revealed to be eight times faster than the PC, achieving an average classification accuracy of over 80%. Lately, EBCI systems-based EEG classifiers have been implemented into the NJT2 board, taking advantage of the NVIDIA^®^ Jetson™ board deployment [33]. In fact, Khatwani et al. [34] implemented a convolutional neural network model into Artix-7 FPGA and NJT2 platforms to detect artifacts carried in EEG signals of multiple channels. Based on the basic ICA algorithm, their method achieved an average accuracy of 74 %, detecting seven different artifact types using 64 EEG channels. In another recent framework [35], convolutional stacked auto-encoder and convolutional long short-term memory models were proposed to classify MI–EEG signals for drone control using the NJT2 board. A latency time of 10 ms was reported for generating drone navigation commands based on left-hand and right-hand imagined movement. Similarly, Ascari et al. [36] implemented a networked nodes modular architecture hosted on the NJT2 platform for outdoor portability. The average accuracy of 50% was achieved based on the subject-specific classification processing EEG signals from Cz, Pz, and {Cz, Pz} channels with an average offset between streams of 0 ±0 ms.

On the other hand, the EEGNet has been implemented more frequently on FPGA boards than on other platforms for EBCI-based EEG signals in the recent literature [37]. Moreover, Hernandez-Ruiz et al. [38] implemented an EEGNet-based architecture into an FPGA board to classify MI–EEG signals, achieving accuracies of 83.15%, 75.74%, and 65.75% for the defined tasks. Lately, Enériz et al. [39] utilized the Xilinx Zynq FPGA to set up a real-time EEGNet-based BCI. Table 1 summarizes the recent state-of-the-art focused on related works.

Finally, regarding the recent literature based on HaLT’s dataset [40], Yan et al. [41] used the referred public dataset to improve classification accuracy by designing an attention mechanism and global features aggregation based on deep learning. They reported an average accuracy of 76.7% for classifying EEG signals of twelve subjects with the EEGNet network. In another work, Keerthi Krishnan and Soman [42] proposed a variational mode-decomposed EEG-spectrum image model for MI classification using the dataset provided by [40]. Their work achieved an average accuracy of 90.2 ± 4.34% with the EEGNet network converting EEG signals from C3, Cz, and C4 channels into spectrum images by using the variational mode decomposition (VMD) and the short-time Fourier transform (STFT). Likewise, a generative adversarial network (GAN) was proposed by An et al. [43] to denoise MI-EEG signals using the same dataset. Lately, the EEGNet network has been implemented to classify MI–EEG signal-based BCI utilizing HaLT’s benchmark [44]. An average classification accuracy of 80.9 ± 8.6% was achieved by classifying EEG signals from eight channels. In sum, taking advantage of more than five BCI interaction paradigms, Kaya’s dataset offers a wide range of BCI implementation possibilities to the related literature. Table 2 presents Kaya’s experiment’s data organization related to six mental imagery tasks. The referred BCI interaction paradigm contemplates 6 MI tasks executed by 12 subjects, each with a determined number of sessions.

## 3. Materials and Methods

The method developed in this work addresses the practical challenge of multi-class classification and expedited processing of EEG signals on dedicated platforms using the NJT2 development board and the artificial neural network EEGNet. All developed processing algorithms are integrated directly into the NJT2 embedded platform to exploit hardware resources.

### 3.1. Overall Flowchart

**Figure 1:**
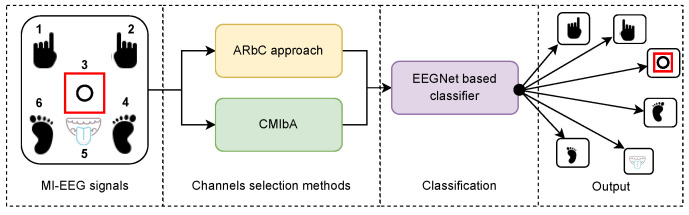
The proposed method overall flowchart. EEG signals of six MI tasks are provided by [40]. The red rectangle centered on the circle refers to “Passive” and moves according to the subject’s MI task. The first step consists of selecting discriminant channels from the 19 provided. Next, two comparative methods are used: the ARbC method and the CMIbA. Therefore, the EEGNet network classifies the feature signals into six classes to give the output.

Figure 1 presents the high-level general diagram of the proposed method. Two main steps are developed to process MI–EEG signals. The first one aims to select discriminant channels employing two approaches (ARbC and CMIbA), while the second implements the EEGNet network to classify discriminant channel features. The ARbC approach also utilizes the EEGNet architecture but with parameters adapted to single-channel signals.

### 3.2. Referred Public Dataset

The dataset published in [40] was used to implement the proposed method. Explicitly, this work used EEG data provided by the BCI interaction paradigm related to six mental imagery states. On a Graphical User Interface (eGUI), a fixation point considered the neutral starting point for tasks was presented to experiment participants. Each trial began with an action signal to imagine movements of the right and left hands, closing and opening the respective fist once, movements of the right and left leg briefly, and movements of the tongue or a circle as a passive response for 1.0 s. For example, the tongue MI task was interpreted as the imaginative pronunciation of a distinct letter as “el”. At the same time, participants did not engage in any voluntary mental imagery until the subsequent trial began for the passive state. These visual stimuli were presented on the eGUI once to the participants in each trial and in sequential order, as presented in Table 3.

A total of 29 recording sessions were performed by seven males and five females aged between 20 and 35 who were declared healthy for the experiment. Each session contains a sequence of BCI interaction segments recorded with a break of 2.0 min, and each trial requires an average of 3.0 s. Accordingly, this BCI interaction contains 87 interaction segments for all 29 sessions in the referred dataset.

**Figure 2:**
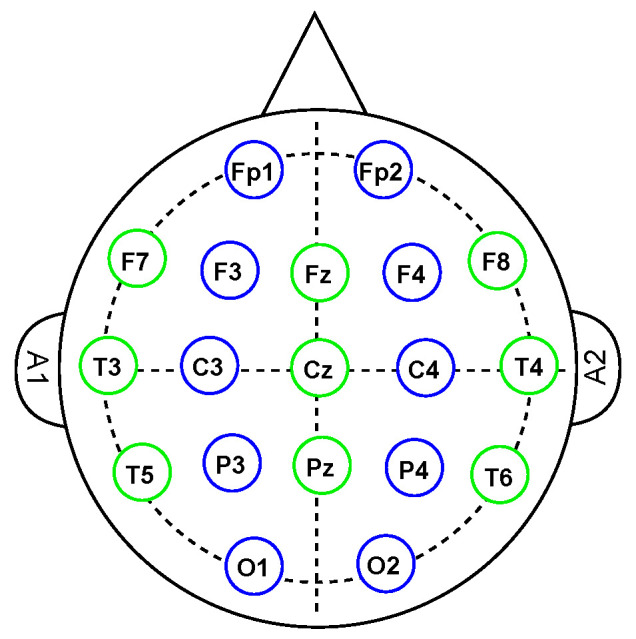
Channels’ spatial location on the skull in making the referred dataset. According to the 10–20 system, uppercase letters define the brain cortex where an electrode is placed. F for Frontal, T for temporal, P for parietal, and O for occipital cortex. The lowercase ”z” is utilized to locate electrodes on the skull’s longitudinal axis. A1 and A2 mean left and right reference voltage electrodes, respectively.

MI–EEG signals were recorded using the EEG-1200 JE-921A standard medical equipment. A total of 19 EEG channels placed according to the standard 10–20 electrodes placement system (see Figure 2) provided the benchmark EEG signals.

**Figure 3:**
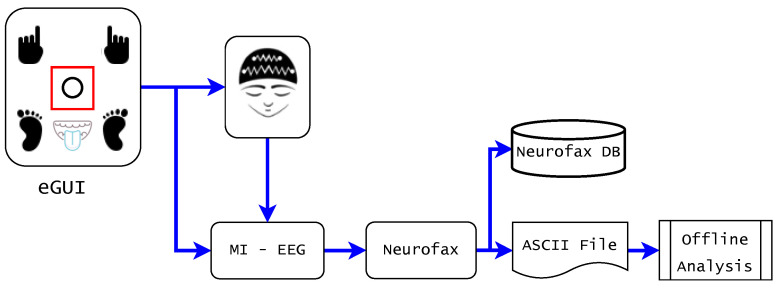
Overview of the EEG acquisition and processing in the experimental paradigm. The red rectangle on the eGUI moves over the specific limb icon as a visual stimulus to engage the respective mental task of imagined movement. MI–EEG signals from six mental states were recorded by EEG-1200 equipment and processed using Neurofax recording software [40]. In addition, ASCII data were converted into Matlab files for further processing.

The Neurofax software was used to record data at 200 Hz, and hardware pass-band filters of 0.53–70 Hz were applied to all recorded EEG signals. It is worth mentioning that the EEG-1200 equipment integrates a hardware notch filter at 50 or 60 Hz to isolate EEG signals from electrical grid interference. Figure 3 presents the experimental paradigm’s data acquisition and processing overview.

### 3.3. NVIDIA Jetson TX2 Embedded Board

The NJT2 is a power-efficient embedded computing device mainly designed for artificial intelligence applications. Building around an NVIDIA Pascal^TM^-family GPU with 8 GB and 59.7 GB/s of memory and bandwidth, respectively, this supercomputer on a module integrates a wide range of standard hardware interfaces. It is also considered a fast and power-efficient platform for robust data applications; the NJT2 card has been used successfully in recent research [34,35,36].

The NVIDIA SDK manager based on Ubuntu is the operating system used on the NJT2 card, accessible from [45]. After installing the operating system, a host computer must load the modules into a Micro-SD card following the steps provided in [46]. Once the Jetson software with the SDK Manager is installed, the NJT2 card is ready to be used as an embedded computer. Additionally, the specific libraries are installed according to the application requirements. Table 4 summarizes the main characteristics of the NJT2 card used to implement the present project, according to the serial number provided.

### 3.4. The EEGNet Network Architecture

**Figure 4:**
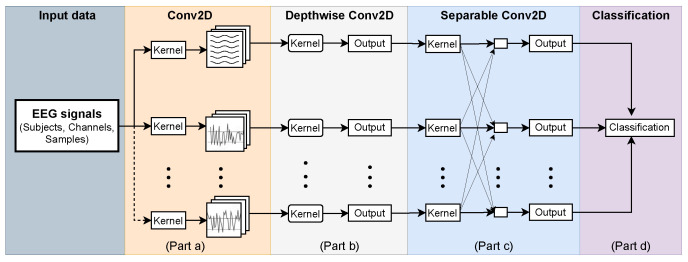
The encapsulated EEGNet structure. EEG signals were organized by subject, channel, and sample length. This data matrix was expanded to four dimensions fulfilling the EEGNet input matrix dimension. In Part (a), temporal features are extracted by Conv2D, and in Part (b), spatial filters are applied to enhance feature maps. Then, feature maps are combined in Separable Conv2D (Part (c)), providing the output class probability (Part (d)).

EEGNet is a compact convolutional network proposed by Vernon et al. [26]. It demonstrated its effectiveness in processing EEG signals for BCI-based systems, considering the numerous related works [47,48,49]. Three convolutional layers are configured in the EEGNet. EEG raw data are first convolved in the temporal layer (Part a) using frequency filters, as shown in Figure 4.

Next, EEG feature maps extracted from the temporal convolutional layer (Part (a)) serve as input for the depthwise convolutional layer (Part (b)), where frequency-specific spatial filters are applied to each feature map. Finally, the separable convolution layer (Part (c)) combines the depthwise and pointwise convolutions of feature maps, both individually and together, to provide an optimal classification (Part (d)). The depthwise and separable convolution layers are activated by the Exponential Linear Unit (ELU) function, defined by

(1) $$f\left({\vec{x}}_{i}\right)=\left\{\begin{array}{cc} x_{i} & \mathrm{for}\;x\geq 0,\\ e^{x_{i}}-1 & \mathrm{otherwise}, \end{array}\right.$$

while the output dense layer uses the Softmax activation function,

(2) $$\sigma \left({\vec{x}}_{i}\right)=\frac{e^{x_{i}}}{\sum_{j=1}^{N}e^{x_{j}}},\;\forall \;\vec{x}={\left[x_{1},x_{2},\ldots ,x_{N}\right]}^{⊺},$$

to predict the output probability of sequence ${\vec{x}}_{i}$ to be classified in class N. Therefore, Equation (2) is considered a normalized probability distribution of output feature sequences. Consequently, an important key for implementing EEGNet is the number of filters for each layer and the kernels’ length. Table 5 shows the EEGNet’s input parameters.

### 3.5. Data Processing

Subjects and channels provide EEG data from the referred benchmark. The number of samples was set to 170, corresponding to the duration of 0.85 s per task, remembering that dataset signals were recorded at 200 Hz. This allowed the removal of artifacts at the beginning and the end of each task signal. Therefore, the first signal processing step consists of channel discrimination to constitute contributing channel subsets. Two strategies were implemented to select the discriminant channels among the 19 provided. The ARbC approach uses the EEGNet network to classify signals of each channel, aiming to constitute the subset of six and eight channels with higher classification accuracy. In contrast, CMIbA utilizes the channels’ mutual information to evaluate how different the cross-entropy measurement value is. The channel selection by the above-mentioned methods was made on the mixed signals of all 12 subjects, i.e., considering signals of the whole dataset. In fact, the constituted discriminant channel subsets can be more suitable for any subject considered separately and be served for the subjects’ performance comparison purposes.

Thus, the ARbC method aims to increase the amount of useful training data allowing the neural network to learn more discriminating features. In fact, the proposed software-level approach uses a group-utility metric-based channel selection strategy to improve classification accuracy [50,51]. Hence, the EEGNet network was configured by setting temporal filters (F1), pointwise filters (F2), and spatial filters (D) to four. This EEGNet filter value choice was made according to preliminary training tests to find the classifier’s optimal configuration according to data features. The model was compiled with the categorical cross-entropy loss function, and the Nadam optimizer was set to 0.001. The network was trained with 2000 epochs, with a batch size of 330, using 10-fold cross-validation. Consequently, two subsets of six and eight discriminant channels were formed.

According to information theory, the mutual information between two random variables σ and ρ is given by

(3) I(σ,ρ)=K(σ)+K(ρ)−K(σ,ρ),

where *K* represents the complexity of information carried by each variable. In the case of probabilistic variables, (3) can be written as

(4) I(X,Y)=H(X)+H(Y)−H(X,Y),

where *H* is the self-information entropy. Based on the assumption that independent random variables should not share mutual information, Kullback–Leibler Divergence (KLD) was used to assess how far a joint distribution of channel signals is from the distribution of their products.

Let P and Q be two probability distributions on the finite channel set S=[1,i,⋯,j,⋯,19], clustering channels signals of the *n*th subject. KLD, or the relative entropy between P and Q, is given by

(5) $$\mathrm{KLD}\left(P\;||\;Q\right)=\sum_{a\;\in \;S}P\left(a\right)\log\frac{P(a)}{Q(a)},$$

where P(a) is the occurrence probability of the *a*th datum. Therefore, mutual information is found evaluating the KLD as,

(6) $$\begin{array}{cc} I(S_{i}\;;\;S_{j}) & =\mathrm{KLD}(P\left(S_{i}\;,\;S_{j}\right)\;||\;P\left(S_{i}\right)P\left(S_{j}\right)), \end{array}$$

where $P(S_{i})$ and $P(S_{j})$ represent signal distributions of channels *i* and *j*, respectively, and $P(S_{i}\;,\;S_{j})$ is a joint distribution. Equation (6) was computed by considering a given channel and its neighbors, two by two, then by pair grouping, based on channel individual distribution to obtain the discriminating channels subset.

- If $S_{i}$ and $S_{j}$ are independents,
  (7) P(a,b)=P(a)P(b).
  Therefore,
  (8) $$\mathrm{KLD}\left(P(a)\cdot P(b)||(P(a)\cdot P(b)\right)=0.$$
- If $S_{i}$ = $S_{j}$,
  (9) $$\begin{array}{cc} I(S_{i}\;;\;S_{i}) & =\sum_{a\;\in \;S}S_{i}\left(a\right)\log\frac{S_{i}\left(a\right)}{S_{i}{\left(a\right)}^{2}}\\ \; & =\sum_{a\;\in \;S}S_{i}\left(a\right)\log\frac{1}{S_{i}\left(a\right)}=H\left(S_{i}\right), \end{array}$$
  where *H* is the self-entropy distribution. Entropy values of two-by-two channel combinations are calculated, that is, the entropy of 171 combinations considering 19 channels. Next, channel combinations with entropy values different from zero are combined with the remaining channels to constitute discriminating channel groups. This process is repeated until a group of *n* channels with the same self-entropy distribution is constituted. Finally, the Discriminant Channel Subset (DCS) is constituted as follows,
  (10) DCS=[1,⋯,n],∀n≤19&DCS⊂S,
  where *n* is the *n*th discriminant channel for all subjects’ signals.

In the next stage, signals of discriminant channel subsets were processed by configuring the EEGNet with new parameters in Keras and TensorFlow, as shown in Table 6. New parameter configuration changes took into account the number of channels, the optimization of hyperparameters, and the learning acceleration at the software level.

EEG data were arranged as a four-dimension tensor to meet the EEGNet’s input dimension [26], receiving the number of samples, the number of channels, the length of the sample, and the unitary position by the input layer. Parameter k in Table 6 refers to the number of channels, taking a value of six or eight depending on the channel discriminant set. The proposed architecture was configured with four temporal filters (F1) in the Conv2D convolutional layer, using 16 parameters for k set to six or eight. After the batch normalization, the Depthwise Conv2D layer activated by the ELU function uses 96 or 128 parameters depending on the discriminating set to learn spatial filters in the temporal convolution, setting the number of spatial filters (D) to 4. For its part, the separable Conv2D layer was configured with 16 pointwise filters (F2), and 512 parameters were used to learn within each kernel length. Both EEGNet configurations for the channel selection and processing steps were compiled and trained into the NJT2 board using a batch size of 330, a categorical cross-entropy loss function, and the Nadam optimizer set to 0.0001. The CLR algorithm with a triangular window was also set between $10^{-6}$ and $5\times 10^{-2}$ to accelerate the learning process by training the EEGNet model with a low number of epochs. Thus, the EEGNet model in the classification stage was trained with 1500 instead of 2000 epochs, using 10 repetitions to validate the results.

## 4. Numerical Results

The k-fold cross-validation method was used both in the channel selection and processing steps to validate the achieved results. Therefore, numerical results were obtained by setting k to 10, meaning that the dataset was repeatedly partitioned into ten subsets, where nine were used for training and one for testing each *k*th iteration. This validation method allows for checking that the model is efficient for different randomized inputs or for some data streams, nothing else. In the channel selection steps, for the ARbC method and CMIbA, training and test sets were formed from signals of all subjects, using nine for training and one for testing. Once the sets of discriminating channels have been constituted, the classification process is performed by exploiting the signals of each subject, taken individually. The proposed model was evaluated using the classification metric given by

(11) $$Accuracy=\frac{TP+TN}{TP+TN+FP+FN},$$

where TP corresponds to true positive when k features are correctly assigned to class *K*, TN means true negative when m features of other classes than *K* are unassigned to class *K*, and FP as false positive are all features erroneously classified into class *K*. Additionally, the confusion matrix metric was used to evaluate the implemented classifier performance discriminating MI tasks.

### 4.1. Channel Selection Results

Processing EEG signals of all subjects by channel, higher classification accuracies were obtained in the order reported in Table 7. Hence, discriminant channel subsets for all the subjects were formed by combining signals of the channel, providing higher accuracy than those of the seven remaining channels, delivering the best accuracies. In the case of P4 and O2 channel selection giving the same classification accuracy (36.7%), tests revealed reliable accuracies in adding the P4 channel to the seven discriminant channels already constituted instead of the O2 channel.

Meanwhile, the channel mutual information approach allowed the formation of six and eight discriminant channel subsets, as presented in Table 8. The number of discriminant channels was determined according to the algorithm proposed in [47], where 6 discriminant electrodes were chosen among the 19 available. In addition, the same subjects participated in the paradigm explored in [47] that was presented in this work, where EEG signals were recorded with the same equipment. Concisely, channel combination tests revealed reliable classification accuracy for subsets of six and eight discriminant channels.

**Figure 5:**
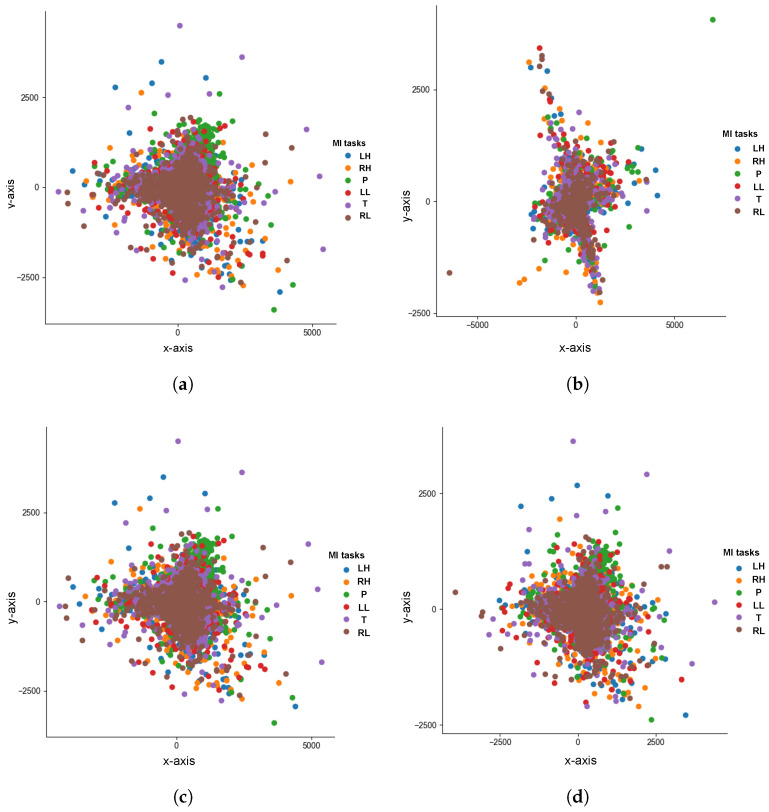
t-SNE distribution illustrations of selected channels’ signals for all subjects using the ARbC method and CMIbA before the main processing step. All figures were plotted in 2-D embedded space using the Euclidean metric, setting the nearest neighbors’ number at 10, the number of iterations for the optimization at 1000, and the gradient norm at 0.0001. (**a**) ARbC: six-channel combination: distribution of {Fp1,F8,Fp2,F7,P3,Cz} channel signals, (**b**) CMIbA: six-channel combination: distribution of {P4,T6,T3,P3,F4,O2} channel signals, (**c**) ARbC: eight-channel combination: distribution of {Fp1,F8,Fp2,F7,P3,Cz,O1,P4} channel signals, (**d**) CMIbA: eight-channel combination: distribution of {P4,T6,T3,P3,F4,O2,Fp2,Fz} channel signals.

The EEG data point distribution was explored using a t-distributed Stochastic Neighborhood Embedding approach (t-SNE) [52] to visualize data clusters according to the class labels. In the case of multi-class EEG data, t-SNE distributions help to visualize high dimensional data considering the nonlinear relationship between features and targeted classes. Therefore, Figure 5 shows the EEG data clusters after selecting six and eight discriminant channels using the ARbC method and CMIbA.

Therefore, only MI–EEG signals from discriminant channel subsets were processed to evaluate the proposed method’s performance.

### 4.2. Results Processing Discriminant Channel Signals

From a general point of view, the results obtained by developing the ARbC method and CMIbA revealed differences considering achieved accuracies and the taxonomy of discriminant channels. The channel selection methods developed refer to whole dataset signals. Table 7 presents average accuracies using the ARbC to classify all dataset signals by channel. According to the ARbC selection algorithm, the eight high-accuracy values were obtained with Fp1, F8, Fp2, F7, P3, Cz, O1, and P4 channel signals, in this order, respectively. For its part, CMIbA allowed the forming of a discriminant channel subset by selecting P4, T6, T3, P3, F4, O2, Fp2, and Fz channels. Therefore, {Fp1,F8,Fp2,F7,P3,Cz,O1,P4} and {P4,T6,T3,P3,F4,O2,Fp2,Fz} discriminant channel subsets were constituted from the 19 provided, proceeding by the ARbC method and CMIbA. Both approaches have the Fp2, P3, and P4 channels in common, considering the subset of eight discriminant channels, while five of those are different. The difference in the taxonomy of channel subsets is explained by the particularity of metrics used by the ARbC method and CMIbA, and also by the signal spread of each channel when mixed with data from other channels.

The results of processing MI–EEG signals from the discriminant channel subset are shown in Table 8. Next, the signals of the selected channels per subject are processed; subject A performed EEG data classification, achieving 86.8% and 89.0% accuracy with the ARbC method and CMIbA, respectively. For its part, subject B achieved an accuracy of 68.0% using the ARbC method using data from eight discriminant channels, compared to 76.3% with CMIbA. For all subjects, increasing the number of discriminant channels revealed improvements in classification accuracy, except for subject K using the ARbC method. According to Table 7, adding two more discriminant channels to subject H using the ARbC method decreased the classification accuracy compared to other subjects. The same observation is made for subject J. The best accuracy was achieved by subject J combining eight discriminating channels with CMIbA (99.7%), while the lower accuracy of 53.7% was obtained using subject I, processing six channel signals.

Finally, concerning the classification accuracy per MI task, Table 9 summarizes the confusion matrix average results by classifying each mental imagery task. Confusion matrices diagonal results reported in the aforementioned table represent the coincidence percentage between the predicted and the true labels for a given output data sequence.

**Figure 6:**
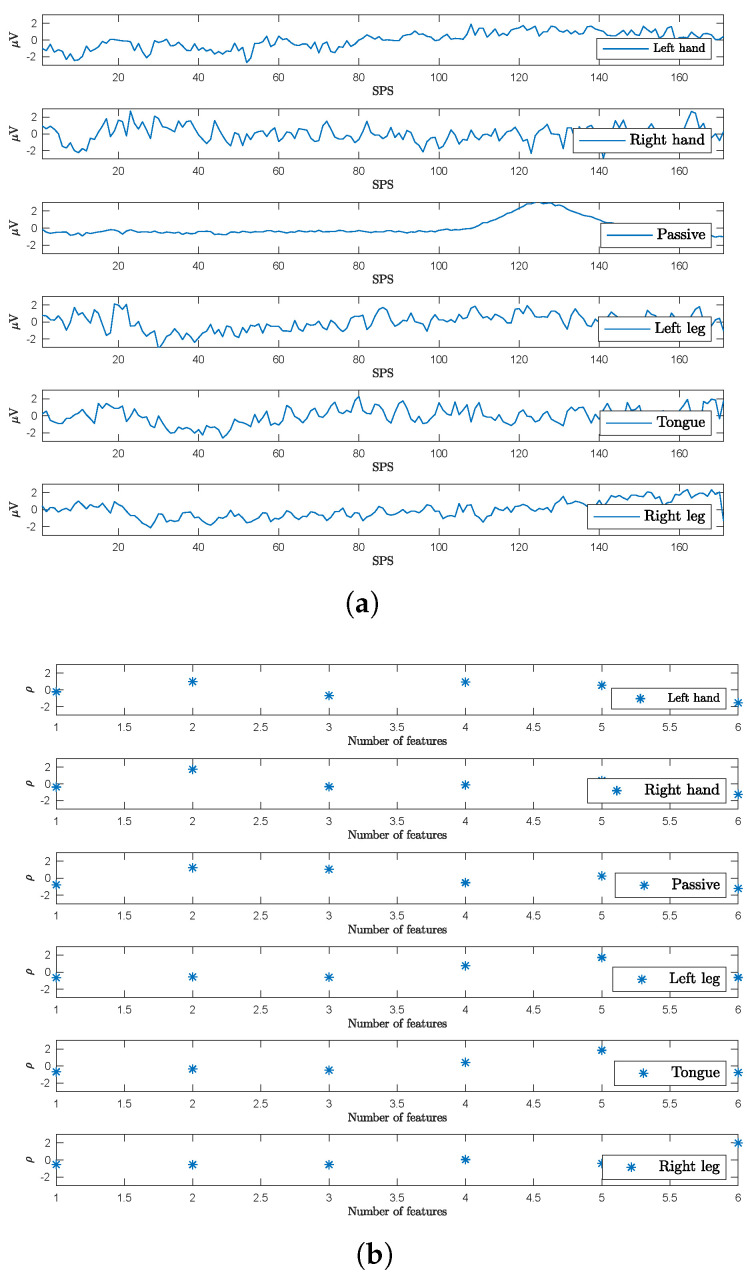
Illustration of MI–EEG features before and after the classification using the EEGNet network. In this example, subject J’s data are provided by the Fp1 channel. The window was set to 170 samples, corresponding to task duration. The normalized magnitude is given in μV, while SPS and ρ mean the number of samples per second and feature magnitude, respectively. (**a**) MI–EEG signals before classification. (**b**) MI–EEG features after classification.

For illustration purposes, Figure 6 presents EEG data related to the described imagined movements for subject J’s Fp1 channel signals. It can be observed that signals corresponding to the passive task are relatively close to magnitude zero before classifying.

## 5. Discussions

Two EEG channel-selection methods are evaluated on how each affects the classification accuracy by increasing the number of channels, considering the same test subject and network architecture. Regarding the cerebral cortices’ spatial activation and for all database signals, almost all brain areas are activated during the experience paradigm. This behavior does not mean that a particular subject would not have had a more activated cortex than others, only that channels were selected based on all subjects’ signals. Further, classifying the set of signals as indicated in Table 8 was carried out illustratively to provide information on the classifier’s average performance (59.3% and 55.2%). However, practically, a BCI system can exclusively be used by one subject at a time; what matters more is each subject’s performance. The results demonstrate that one selection approach can be more effective than the other, depending on the EEG data provided by each subject and on the number of channels.

For subjects K and M, the ARbC method is efficient. In contrast, the CMIbA is suitable for subjects A, B, C, E, F, G, H, I, J, and L. For subjects, C, E, G, H, I, J, and M, either the ARbC method or CMIbA may be recommended depending on the number of discriminating channels. For six discriminant channels, the ARbC method is suitable, while for eight discriminant channels, the CMIbA is desirable.

Regarding classification accuracies, results achieved in this work are compared to those published in the recent related works, as presented in Table 10. In [42], a VMD mode approach to extract EEG features was implemented before using the EEGNet in the classification step. Their work also implemented a subject-dependent classification approach using the referred dataset. Comparing their results with those achieved in this work, subjects A, C, J, and L performed data classification, while the remaining subjects obtained the best results with the approach developed in [42]. This difference in the accuracy evaluation is essentially due to the implemented strategies in the preprocessing before classifying EEG signals. Lately, Yan et al. [41] proposed a similar work based on Kaya’s benchmark. They reported an average accuracy of 76.79% in classifying MI-EEG signals from 19 channels. This work achieved an average accuracy of 83.7% using eight channel features.

Focusing on the processing unit and the latency, another aspect targeted in this work, Table 11 presents the latency per MI task per subject. The lower average latency of 36.7 ms was obtained by subject J while classifying MI tasks; because of the low number of subject J’s sessions.

Therefore, Table 12 compares this framework with similar works in the recent literature. The purpose is to compare EEGNet network successful implementations on the NJT2 board with the proposed method. Khatwani et al. [34] achieved a latency inferior to 84.1 ms using 64 EEG channels to detect an artifact type. The maxima latency was evaluated at 84.1 ms classifying EEG artifacts. In this work, the average latency per task and per subject was evaluated at 48.7 ms. For their parts, Maiti et al. [35] controlled a drone generating commands with a maximum latency of 10 ms. From a particular point of view, this latency improvement is essentially due to the few channels, compared to the number of channels used in this work. In another work, Ascari et al. [36] processed EEG signals with an average latency of 0 ± 0 ms using two channels. Despite the size of the datasets used in the above-mentioned works, the number of channels used is a determinant factor in evaluating the latency per MI task.

Therefore, this framework uses robust EEG data provided by twelve subjects in comparison to the mentioned works. Each MI task needed 48.7 ms to be classified, processing signals from eight discriminant channels. Only 7.6% of the proposed method’s NJT2 resources were used.

## 6. Conclusions

This work developed a multi-class classification of MI–EEG signals for BCI systems, implementing EEGNet on the NJT2 platform. Prior to processing signals, two channel-selection approaches were used to determine the discriminant channel subsets, the ARbC approach, and CMIbA. Since discriminant channel subsets were made up, the EEGNet classified MI–EEG signals into six classes. The results obtained prove the classification accuracy improvement using the two proposed channel selection approaches. Increasing the number of channels allowed one approach to achieve more reliable accuracies than the other approach, depending on the subject data. Processing acceleration strategies implemented by utilizing the NJT2 platform resources and the CLR algorithm allowed for dealing with the processing time challenge. The highest classification accuracy of 99.7% was achieved with subject J’s signals, processing data with a latency of 36.7 ms per task. The successful carrying out of the classifier presented in this work is offered as an alternative for the embedded BCI system’s development. However, based on the approaches developed in this work, increasing the number of discriminating channels beyond eight tends to decrease the classification accuracy. In future work, we expect to control an electric car using the results achieved in this work. Moving forward, backward, turning right and left, neutral, and accelerating are the expected tasks to be performed. The framework’s source codes are available from 1 January 2023, on GitHub https://github.com/Tatyvelu/Motor-Imagery-Multi-Tasks-Classification-for-BCIs-Using-the-Jetson-TX2-board-and-a-Modified-EEGNet-A.

## Figures and Tables

**Table 1:** The state-of-the-art summary of related works. Ch means the number of channels.

Works	Platform	Dataset	Ch	Latency
				per Task
Khatwani et al. [34]	NJT2	Own	64	≤84.1 ms
Maiti et al. [35]	NJT2	BCI competition IV	3	9–10 ms
Ascari et al. [36]	NJT2	Own	2	0 ±0 ms

**Table 2:** Summary of BCI interaction paradigm data related to six mental imagery tasks, as presented in [40].

No.	Subject	Classes	Sessions	Samples
1	A	6	3	2877
2	B	6	3	2869
3	C	6	2	1916
4	E	6	3	2855
5	F	6	3	2879
6	G	6	3	2867
7	H	6	2	1912
8	I	6	2	1836
9	J	6	1	946
10	K	6	2	1914
11	L	6	2	1904
12	M	6	3	2866
13	All	6	29	27,641

**Table 3:** The BCI interaction segment for imagining limbs motion, following the eGUI’s visual stimuli.

Relaxation	1⟶	2⟶	3⟶	4⟶	5⟶	6⟶
	Left hand	Right hand	Passive	Left leg	Tongue	Right leg

**Table 4:** NVIDIA Jetson TX2 main characteristics and resources.

Label	Characteristics
NJT2 board	Serial 0320218091017, model 699-82597-0000-501 C
GPU	256-core NVIDIA Pascal^TM^ GPU architecture with 256 NVIDIA CUDA cores
CPU	Dual-Core NVIDIA Denver 2 64-Bit CPU Quad-Core ARM^®^ Cortex^®^-A57 MPCore
Memory	8 GB 128-bit LPDDR4 Memory 1866 MHx—59.7 GB/s
Storage	32 GB eMMC 5.1
Computing capacity	1.33 TFLOPs
Power consumption	7.5 W/15 W
Mechanical	69.6 mm × 45 mm, 260-pin edge Connector
Networking	10/100/1000 BASE-T, 802.11ac WLAN, Bluetooth

**Table 5:** Configurable input parameters of the EEGNet network, modified from [26].

Parameters	Descriptions
nb_classes	Number of classes to classify
Chans	Number of channels
Samples	Number of EEG data time points
DropourRate	Dropout fraction
kerneLength	Length of temporal convolution in the first layer (Conv2D).
F1, F2	Numbers of temporal filters (F1) and pointwise filters (F2) to learn.
D	Number of spatial filters to learn within each kerneLength
dropoutType	Either SpatialDropout2D or Dropout

**Table 6:** EEGNet parameters for processing *k* discriminant channel signals. This study used k=6 and k=8 discriminant channels.

Layer (Type)	Output Shape	Parameters
Input Layer	(None, k, 170, 1)	0
Conv2D	(None, k, 170, 4)	16
Batch_normalization_1	(None, k, 170, 4)	16
Depthwise_conv2D	(None, 1, 170, 16)	96/128
Batch_normalization_2	(None, 1, 170, 16)	64
Activation_1	(None, 1, 170, 16)	0
Average_pooling2D_1	(None, 1, 42, 16)	0
Dropout_1	(None, 1, 42, 16)	0
Separable_conv2D	(None, 1, 42, 16)	512
Batch_normalization_3	(None, 1, 42, 64)	64
Activation_2	(None, 1, 42, 16)	0
Average_pooling2D_2	(None, 1, 5, 16)	0
Dropout_2	(None, 1, 5, 16)	0
Flatten	(None, 80)	0
Dense	(None, 6)	486
Softmax	(None, 6)	0

**Table 7:** Achieved classification accuracies by implementing the ARbC approach to constitute discriminating channel sets. The highest accuracy is highlighted in blue, while the seven highest accuracies are shown in boldface.

Ref.	Channel	Brain Area	Accuracy (%)
1	Fp1	Frontal (attention)	39.5
2	Fp2	Frontal (Judgment restrains impulses)	**39.1**
3	F7	Frontal (Verbal expression)	**38.4**
4	F3	Frontal (Motor planning of left-upper extremity)	36.4
5	Fz	Frontal central (Motor planning (midline))	36.4
6	F4	Frontal (Motor planning of left-upper extremity)	35.1
7	F8	Frontal (Emotional expression)	**39.2**
8	T3	Temporal (Verbal memory)	34.7
9	C3	Central (sensorimotor integration (right))	36.5
10	Cz	Central (sensorimotor integration (midline))	**37.0**
11	C4	Central (sensorimotor integration (left))	35.9
12	T4	Temporal (Emotional memory)	35.9
13	T5	Temporal (Verbal understanding)	36.3
14	P3	Parietal (cognitive processing special temporal)	**37.4**
15	Pz	Parietal (cognitive processing)	35.7
16	P4	Parietal (“Math word problems”, “Non-verbal reasoning”)	**36.7**
17	T6	Temporal (Emotional understanding and motivation)	36.4
18	O1	Occipital (visual processing)	**37.0**
19	O2	Occipital (visual processing)	36.7

**Table 8:** Results achieved with the implemented channel selection approaches.

Subject	Channel	Average Accuracies (%) Depending on the Number of Channels			
	**Selection**	**6**	**Accuracy**	**8**	**Accuracy**
A	ARbC	{Fp1,F8,Fp2,F7,P3,Cz}	80.6	{Fp1,F8,Fp2,F7,P3,Cz,O1,P4}	86.8
	CMIbA	{P4,T6,T3,P3,F4,O2}	86.5	{P4,T6,T3,P3,F4,O2,Fp2,Fz}	89.0
B	ARbC	{Fp1,F8,Fp2,F7,P3,Cz}	63.9	{Fp1,F8,Fp2,F7,P3,Cz,O1,P4}	68.0
	CMIbA	{P4,T6,T3,P3,F4,O2}	68.7	{P4,T6,T3,P3,F4,O2,Fp2,Fz}	76.3
C	ARbC	{Fp1,F8,Fp2,F7,P3,Cz}	89.1	{Fp1,F8,Fp2,F7,P3,Cz,O1,P4}	90.9
	CMIbA	{P4,T6,T3,P3,F4,O2}	83.0	{P4,T6,T3,P3,F4,O2,Fp2,Fz}	92.2
E	ARbC	{Fp1,F8,Fp2,F7,P3,Cz}	76.6	{Fp1,F8,Fp2,F7,P3,Cz,O1,P4}	78.3
	CMIbA	{P4,T6,T3,P3,F4,O2}	70.8	{P4,T6,T3,P3,F4,O2,Fp2,Fz}	82.5
F	ARbC	{Fp1,F8,Fp2,F7,P3,Cz}	71.6	{Fp1,F8,Fp2,F7,P3,Cz,O1,P4}	79.2
	CMIbA	{P4,T6,T3,P3,F4,O2}	72.4	{P4,T6,T3,P3,F4,O2,Fp2,Fz}	80.4
G	ARbC	{Fp1,F8,Fp2,F7,P3,Cz}	84.0	{Fp1,F8,Fp2,F7,P3,Cz,O1,P4}	86.0
	CMIbA	{P4,T6,T3,P3,F4,O2}	81.9	{P4,T6,T3,P3,F4,O2,Fp2,Fz}	87.3
H	ARbC	{Fp1,F8,Fp2,F7,P3,Cz}	57.0	{Fp1,F8,Fp2,F7,P3,Cz,O1,P4}	57.8
	CMIbA	{P4,T6,T3,P3,F4,O2}	56.2	{P4,T6,T3,P3,F4,O2,Fp2,Fz}	65.5
I	ARbC	{Fp1,F8,Fp2,F7,P3,Cz}	56.4	{Fp1,F8,Fp2,F7,P3,Cz,O1,P4}	57.6
	CMIbA	{P4,T6,T3,P3,F4,O2}	53.7	{P4,T6,T3,P3,F4,O2,Fp2,Fz}	67.9
J	ARbC	{Fp1,F8,Fp2,F7,P3,Cz}	99.6	{Fp1,F8,Fp2,F7,P3,Cz,O1,P4}	99.5
	CMIbA	{P4,T6,T3,P3,F4,O2}	98.8	{P4,T6,T3,P3,F4,O2,Fp2,Fz}	99.7
K	ARbC	{Fp1,F8,Fp2,F7,P3,Cz}	83.0	{Fp1,F8,Fp2,F7,P3,Cz,O1,P4}	79.4
	CMIbA	{P4,T6,T3,P3,F4,O2}	76.8	{P4,T6,T3,P3,F4,O2,Fp2,Fz}	79.3
L	ARbC	{Fp1,F8,Fp2,F7,P3,Cz}	85.7	{Fp1,F8,Fp2,F7,P3,Cz,O1,P4}	93.9
	CMIbA	{P4,T6,T3,P3,F4,O2}	90.4	{P4,T6,T3,P3,F4,O2,Fp2,Fz}	98.0
M	ARbC	{Fp1,F8,Fp2,F7,P3,Cz}	78.7	{Fp1,F8,Fp2,F7,P3,Cz,O1,P4}	83.7
	CMIbA	{P4,T6,T3,P3,F4,O2}	79.5	{P4,T6,T3,P3,F4,O2,Fp2,Fz}	81.9
{A,B, *…*, M}	ARbC	{Fp1,F8,Fp2,F7,P3,Cz}	55.5	{Fp1,F8,Fp2,F7,P3,Cz,O1,P4}	59.3
	CMIbA	{P4,T6,T3,P3,F4,O2}	52.2	{P4,T6,T3,P3,F4,O2,Fp2,Fz}	55.2

**Table 9:** Summary of confusion matrices’ diagonal results classifying MI tasks separately. The average accuracies per MI task do not include “{A,B, *…*, M}” subjects. The CMIbA was used for that purpose.

Subject	Average Accuracies (%) per MI Task					
	**Left Hand**	**Right Hand**	**Passive**	**Left Leg**	**Tongue**	**Right Leg**
A	80	80	80	80	80	80
B	75	75	75	75	75	75
C	90	90	90	90	90	90
E	75	75	75	75	75	75
F	77	77	77	77	77	77
G	85	85	85	85	85	85
H	67	67	67	67	67	67
I	67	67	67	67	67	67
J	100	100	100	100	100	100
K	80	80	80	80	80	80
L	100	100	100	100	100	100
M	80	80	80	80	80	80
Average	81.3	81.3	81.3	81.3	81.3	81.3
{A, B, *…*, M}	57	57	57	57	57	57

**Table 10:** Comparison with other state-of-the-art methods related to the Halt dataset. Sel.Ch. means selected channels, and μ is the average classification accuracy.

Subject	Works					
	Keerthi et al. [42]		Yan et al. [41]		Proposed Method	
	VMD + STFT + EEGNet		EEGNet		CbA/CbMI + EEGNet	
	Sel.Ch.	Acc.(%)	Sel.Ch.	Acc.(%)	Sel.Ch.	Acc.(%)
A	3	86.74	19	87.40	8	89.0
B	3	97.42	19	67.22	8	76.3
C	3	82.93	19	82.36	8	92.2
E	3	91.84	19	76.94	8	82.5
F	3	94.27	19	70.32	8	80.4
G	3	89.02	19	89.33	8	87.3
H	3	87.25	19	43.46	8	65.5
I	3	90.18	19	44.25	8	67.9
J	3	88.55	19	98.84	8	99.7
K	3	85.76	19	81.03	6	83.0
L	3	92.49	19	95.35	8	98.0
M	3	96.01	19	84.93	8	83.7
μ	–	90.20	–	76.79	–	83.7

**Table 11:** Summary of average latency per subject for classifying each MI task.

Subject	Average Latency (ms) per MI Task					
	**Left Hand**	**Right Hand**	**Passive**	**Left Leg**	**Tongue**	**Right Leg**
A	56.1	56.1	56.1	56.1	56.1	56.1
B	55.7	55.7	55.7	55.7	55.7	55.7
C	42.7	42.7	42.7	42.7	42.7	42.7
E	55.2	55.2	55.2	55.2	55.2	55.2
F	57.2	57.2	57.2	57.2	57.2	57.2
G	55.8	55.8	55.8	55.8	55.8	55.8
H	42.3	42.3	42.3	42.3	42.3	42.3
I	43.8	43.8	43.8	43.8	43.8	43.8
J	36.7	36.7	36.7	36.7	36.7	36.7
K	42.1	42.1	42.1	42.1	42.1	42.1
L	41.8	41.8	41.8	41.8	41.8	41.8
M	56.0	56.0	56.0	56.0	56.0	56.0
Average	48.7	48.7	48.7	48.7	48.7	48.7
{A,B, *…*, M}	135	135	135	135	135	135

**Table 12:** Comparison with related works using neural network architectures on the NJT2 board.

Methods	Platform	Dataset	Number of	Latency
			Channels	per Task
Khatwani et al. [34]	NJT2	Own	64	≤84.1 ms
Maiti et al. [35]	NJT2	BCI competition IV	3	9–10 ms
Ascari et al. [36]	NJT2	Own	2	0 ± 0 ms
Proposed method	NJT2	HaLT [40]	6, 8	48.7 ms

## Data Availability

Data are available under a formal demand.

## Abbreviations

BCI	Brain–Computer Interface
EEG	Electroencephalogram
BCV	Brain–Controlled Vehicle
EOG	Electrooculogram
EMG	Electromyogram
SSVEP	Steady-State Evoked Potentials
MI	Motor Imagery
MI–EEG	Motor Imagery EEG
FPGA	Field-Programmable Gate Arrays
SVM	Support Vector Machines
EEGNet	Compact convolutional neural network for EEG-based BCI
NJT2	NVIDIA Jetson TX2
STFT	Short-Time Fourier Transform
VMD	Variational Mode Decomposition
GAN	Generative Adversarial Network
ARbC	Accuracy Rating-based Classifier
CMIbA	Channels Mutual Information-based Approach
CLR	Cyclic Learning Rate
EBCI	Embedded Brain–Computer Interface
ICA	Independent Component Analysis
PC	Portable Computer
CNN	Convolutional Neural Network
eGUI	Graphical User Interface
ASCII	American Standard Code for Information Interchange
CPU	Central Processing Unit
GPU	Graphics Processor Unit
SDK	Software Development Kit
LPDDR4	Low Power Double Data Rate
eMMC	Embedded Multi-Media Card
TFLOPS	Trillion Floating-Point Operations Per Second
WLAN	Wireless Local Area Network
ELU	Exponential Linear Unit
KLD	Kullback–Leibler Divergence
DCS	Discriminant Channel Subset
t-SNE	t-distributed Stochastic Neighborhood Embedding
LUT	Look-Up-Table
SPS	Samples Per Second
CLR	Cyclical Learning Rate
MFLOPS	Million Floating-Point Operations Per Second
